# Promoters with Cancer Cell-Specific Activity for Melanoma Gene Therapy 

**Published:** 2011

**Authors:** V.V. Pleshkan, I.V. Alekseenko, M.V. Zinovyeva, T.V. Vinogradova, E.D. Sverdlov

**Affiliations:** Shemyakin and Ovchinnikov Institute of Bioorganic Chemistry, Russian Academy of Sciences; Institute of Molecular Genetics, Russian Academy of Sciences

**Keywords:** melanoma, gene therapy, tissue-specific promoters, specific expression of a transgene

## Abstract

Melanoma is one of the most aggressive tumors. It develops from pigment-forming cells (melanocytes) and results in a high number of lethal outcomes. The use of genetic constructs with the ability to specifically kill melanoma cells, but not normal cells, might increase the lifespan of patients, as well as improve their quality of life. One of the methods to achieve a selective impact for therapeutic genes on cancer cells is to utilize a transcriptional control mechanism using promoters that are specifically activated only in cancerous cells. In this review, promoters of the genes that are preferentially expressed in melanoma cells are described. These promoters, and other highly melanoma-specific regulatory elements, reduce the unspecific expression of therapeutic genes in normal tissues. Moreover, cancer-specific promoters and their elements are advantageous for the development of universal anticancer drugs. Examples of the use of double promoters that have a high potential as instruments in cancer gene therapy are also given in this review.

## INTRODUCTION 

A steady increase in deaths caused by malignant skin melanoma has recently been in evidence around the world, including in Russia. Malignant melanoma belongs to the most aggressive variety of tumors; the five-year survival rate of patients is less than 50%. Melanoma especially stands out by its early metastazing; therefore, chemotherapy and radiation therapy are not very effective against the disease [1–3].

In the longer term, an important role could be played by gene therapy methods based on the introduction of a therapeutic gene (transgene) into melanoma cells in patients. Therapeutic contructs may contain genes that compensate for the reduction in the expression of suppressor genes, which results in tumor development. Or conversely, they may contain genes whose products neutralize the increased expression of an undesirable gene (oncogene) [4–7]. The approach in which the so-called suicide genes are used is considered to be one of the most universal strategies among genetic strategies for killing cancer cells [8–10]. In this case, a gene is introduced into a tumor cell; which encodes an enzyme that is not typical for the normal cell and is capable of converting the compound that has no toxicity towards healthy cells (prodrug) into a toxin that results in the killing of tumor cells containing a suicide gene. Thus, the selective killing of cancer cells in which the suicide gene acts is provided [11–13]. The gene encoding the herpes simplex virus thymidine kinase ( *HSVtk* ) and the gene encoding yeast cytosine desaminase ( *FCY1* ) are the most widely known and the most frequently used suicide genes. As opposed to cellular thymidine kinase, HSVtk has the ability to phosphorylate the antiherpes drugs acyclovir and ganciclovir. The cells transformed by the *HSVtk* gene die in the presence of these agents, since cellular kinases convert the phosphorylated acyclovir and ganciclovir into triphosphates, which are inserted into the newly synthesized DNA upon cytokinesis and terminate its subsequent synthesis. It is the dividing cells that die, rather than the resting cells, in which DNA is not synthesized and ganciclovir or acyclovir is not inserted [14]. The gene *HSVtk * has been successfully used for the experimental therapy of numerous types of tumors in animals. A number of systems comprising this gene have been undergoing clinical trials [13, 15, 16]. The use of the *FCY1 * gene is based on the absence of its product, cytosine desaminase, in mammal cells and on its ability to convert 5-fluorocytosine, which is not toxic to humans, into a known cytostatic, 5-fluorouracil [17, 18]. After the incorporation of cytosine desaminase into tumor cells and its specific expression in these cells, the introduction of fluorocytosine into a patient’s organism results in its intracellular conversion into fluorouracil, which reduces the toxic effect that fluorouracil has on normal cells.

The specificity of the vector to tissue of this type can be specified either by delivering it exactly into the tissue or by creating conditions for the specific expression of the transgene in the specified tissue. Due to its simplicity, the latter method has been used more frequently. With this aim, promoters and enhancers that act specifically in the tumors of the given tissue are used in the construction of vectors [19, 20]. In the case of malignant melanoma, promoters and enhancers of the genes that are involved in melanin biosynthesis are used. The fact that transgene expression is ensured only in tumor cells, and not in normal cells, is an advantage of the promoters and other regulatory elements that are strictly specific to tumors of this type. However, their non-universality and the inevitable increase in the cost of drugs based on the promoters connected with this fact is a significant disadvantage of such approaches. A variant which offers a potential compromise consists in the use of more universal tumor-specific promoters capable of acting in a wide range of tumors, but not in normal cells. Although this approach slightly increases the risk of affecting normal tissues, it is considered to be more economically viable, since the same constructs can be used for the therapy of a wide range of tumors. An additional factor that supports promoters having a broader action spectrum is connected with the poorly studied specificity of gene expression in metastases of the given tumor. There is no strict guarantee that a narrow-specific promoter that acts well in a primary tumor will retain this ability in all its metastases. The use of universal promoters reduces the probability of promoter inactivation in metastases.

This review describes the structure and properties of promoters that are specific to melanoma cells and those that are active for a wide range of tumors but are still used in the gene therapy of melanoma. 

## PROMOTERS THAT ARE SPECIFICALLY ACTIVE IN MELANOCYTES 

The most well-studied promoter modules that control the specific expression of the therapeutic gene in melanoma cells, with recent widespread application, are promoters of the tyrosinase gene ( *TYR* ) or the gene encoding the melanoma inhibitory activity ( *MIA* ), sometimes combined with distal elements of other promoters and/or the enhancers [21, 22].

**Tyrosinase gene promoter**

Tyrosinase (TYR) is one of the key enzymes required for the synthesis of pigment melanin, which is formed only in melanocytes and in retinal pigment epithelium. The *TYR* gene is expressed only in the specified cells and in many (but not in all) human melanomas and serves as a good marker of melanocyte differentiation [23]. It has been shown that the 5’-region with respect to the transcription start site (TSS) determines the specificity of tyrosinase gene expression [24, 25]. It was demonstrated by the deletion analysis that the minimal promoter of the human tyrosinase gene is likely to be located in coordinates -209/+51 with respect to TSS [26] ( *Fig. A* ). A 115 bp fragment is enough for a tissue-specific activity of the human *TYR* promoter [24]. This promoter fragment contains three positive regulatory elements: the conservative element that is typical for melanocyte-specific promoters – M-box (-104/-37 from the TSS), linked with nine nucleotides that are known as CR1; the Sp1-site (-45/-37 from the TSS); and the evolutionarily conservative element CR2 consisting of the E-box motif and the octamer element (-14/+1 from the TSS) overlapping with it [24]. It is significant that the octamer element in the *TYR* promoter is degenerate in many mammals, including mice [27]. The E-box contains the CANNTG motif, which binds bHLH family transcription factors (basic-helix-loop-helix). This motif was detected in *TYR* gene promoters in various animal species. A similar motif can be found within the M-box and the enhancer region of the tyrosinase gene [27]. Melanocyte-specific expression of the tyrosinase gene is activated upon the binding of the product of the *MITF* gene to the promoter region, including the M-box and the E-box starting region [24].

Promoters of the human and mouse tyrosinase genes are characterized by a high degree of identity of the nucleotide sequence [25]. However, a functional comparison of the promoters of these genes for a human and a mouse has demonstrated that the human *TYR* promoter has a lower efficacy and specificity of expression in melanocytes in comparison with the mouse *Tyr* promoter [25]. It has been assumed that an enhancer plays a significant role in the activity of the human *TYR* promoter. The human *TYR* enhancer, termed tyrosinase distal element (TDE), is located at positions -2014/-1810 and contains the E-box [25]. The binding of two MITF transcription factors to the E-boxes found within both the promoter and enhancer is significant for the specific activity of the human tyrosinase gene [21].

A 200 bp enhancer identical to the human *TYR* enhancer was also found in the 5’-region of the mouse tyrosinase gene; however, only the promoter is essential for manifesting the specific activity of the mouse gene [26, 28].

**Promoter of the gene encoding melanoma inhibitory activity (MIA)**

The gene encoding the melanoma inhibitory activity ( *MIA* ) is expressed predominately in melanoma or chondrosarcoma cells, in certain adenocarcinomas and chondrocytes; however, it is inactive in normal melanocytes [29–31]. The MIA protein, the secreted inhibitor of cell growth, prevents the attachment of melanoma cells to the extracellular matrix, thus promoting invasion and metastazing [32–34]. As opposed to the *TYR* gene promoter, the activity of the human *MIA* gene promoter correlates with melanoma progression [35]. It has been known that the 1.4 kb-long region flanking the 5’-fragment of the *MIA* gene with respect to the transcription starting site provides the specificity of expression of this gene only in melanoma cells, not in melanocytes [36]. By means of deletion analysis it has been demonstrated that the minimal promoter of the human *MIA* gene consists of 212 bp (positions -211/+1) and of 230 bp (-229/+1), in the case of the mouse *MIA* gene, as is shown in *Fig. B* . The elements of the promoters of this gene for humans and mice, which are responsible for the specificity of expression in melanoma cells, are located at positions -212/-170 and -230/-130, respectively [36, 37]. The structure and size of the human and mouse *MIA* promoters are conservative and contain identical elements, which can differ only in their position [38]. Thus, both *MIA* promoters contain no TATA-box and/or CAAT-sequence near the transcription start site. Site Sp1 is conservative and is located at position -108/-103 in the human *MIA* promoter, and at 106/-101, in the mouse promoter. *MIA* promoters contain multiple E-boxes with bHLH-binding sites [36] ( *Fig. B* ). The binding site of the NF-κB transcription factor is also highly conservative in human and mouse genes; in addition, it is located at different positions (-207/-198 and -819/-811, respectively) [36]. Deletion or mutation of this site results in a considerable decrease in human *MIA* promoter activity in melanoma cell lines [36]. Human and mouse *MIA* promoters also comprise such widespread elements as the binding sites α-INF-2, C/EBP, GATA-1, GM-CSF, NF-IL6, NF-κB, TCF-2, etc. It is of interest that the activity of the *MIA* promoter may be dependent on the NF-κB factor, which controls the expression of the genes encoding the immune response, apoptosis, and cell cycle [36]. At the time of writing, nothing was yet known about the enhancer elements of the *MIA* gene.

**Promoter of the melacortin receptor gene**

The melanocortin 1 receptor (receptor MC1R) is expressed predominately in melanocytes and melanomas [39, 40]. MC1R is a transmembrane G-protein-coupled receptor of the α-melanocyte-stimulating hormone (α-MSH). High expression of the *MC1R * gene is also typical of the cell lines originating from primary and metastatic melanomas [41]. Minor amounts of these receptors are found in other tissues and cells; e.g., in testicles, ovary, adrenal glands, keratinocytes, dendrite cells, and activated monocytes [41, 42]. The 3.2 kb-long fragment located in the 5’-region with respect to the TSS of the *MC1R * gene contains several Sp1-binding motifs, consensus sequences of the AP-1 and AP-2 sites, and several E-boxes. The *MC1R * gene promoter contains no TATA- or CAAT-sequences near the TSS [43, 44]. Melanocyte-specific expression of *MC1R* , similar to that of the tyrosinase gene, is activated upon binding of the MITF transcription factor to the E-box [45–48]. It has been shown that 150 bp located above the ATG codon of the *MC1R * gene are sufficient in order to initiate the melanocyte-specific transcription [49]. This minimal promoter can be considered as one of the possible candidates for the transcriptional control of transgene expression in melanoma cells.

**The use of heterologous regulatory elements to enhance melanoma promoters**

The use of *cis* -regulatory elements based on various combinations of the tyrosinase gene promoter and additional heterologous enhancers, which control transcription for specific transgene expression in melanoma, has been described in a number of studies [21, 22, 26]. It is apparent that the *TYR* promoter ensures high activity and specificity of the human *TYR* gene only in the presence of the enhancer element [25]. The construct consisting of the human *TYR* promoter (209 bp) and two or more sequentially attached human *TYR* enhancers (200 bp each) exhibit the highest specific effect upon transfection of melanoma cell lines [26]. The dimer of the mouse tyrosinase gene enhancer linked with the mouse gene promoter also enhances the activity and specificity of the mouse gene [26]. Similar constructs were used when constructing the conditionally replicating adenoviruses (CRAds), where the promoter of the adenoviral *Е1А* gene was substituted for the promoter hTyr2E/P that is specifically active in melanomas and consists of the dimer of the human tyrosinase gene enhancer and the core promoter of this human gene [50]. The resulting adenoviruses manifested a pronounced oncolytic effect on melanoma cell lines. The cytotoxic effect of these constructs was also comparable in terms of the level of CRAd action with the strong nonspecific cytomegalovirus (CMV) promoter. Meanwhile, a strong decrease in the cytotoxic effect of the adenovirus on normal fibroblasts and keratinocytes was observed [50]. Thus, the use of the specific promoter in the *E1A * region of the adenovirus genome made it possible to attain a selective effect on melanoma cells. The binding of several enhancers of the mouse tyrosinase gene had an even stronger effect on the activity and specificity of the human *TYR* promoter. Thus, when constructing oncolytic adenoviral vectors, the TETP promoter construct was used. It contained the core human tyrosinase promoter (TP) and a tandem consisting of four mouse tyrosinase gene enhancers (tyrosinase enhancer – TE) [51]. The use of this promoter to control the expression of the luciferase reporter gene enhanced the activity of the latter in melanoma cells by several orders of magnitude in comparison with the activity in non-melanoma cells [51]. The replacement of mouse enhancers by human enhancer sequences in these constructs resulted in only a 2–3-fold increase in reporter gene activity [51]. The same promoter (TETP) was used to control the expression of suicide genes that were delivered to melanoma cells by bacteria *Listeria monocytogenes* [52]. It was demonstrated earlier that avirulent listeria strains can penetrate into solid tumor cells and provide replication of the delivered plasmids in them [53–55]. Upon bacterial delivery of plasmids, in which the TETP promoter controls the suicide gene of purine nucleoside phosphorylase (PNP) or the chimeric gene of cytosine desaminase and phosphoryl transferase (FCU1) of yeasts, the transgene is specifically expressed in B16 melanoma cells, but not in kidney COS-1 fibroblasts. If a nonspecific CMV promoter is used, the suicide genes are expressed in both cell lines [52].

**Fig. 1:**
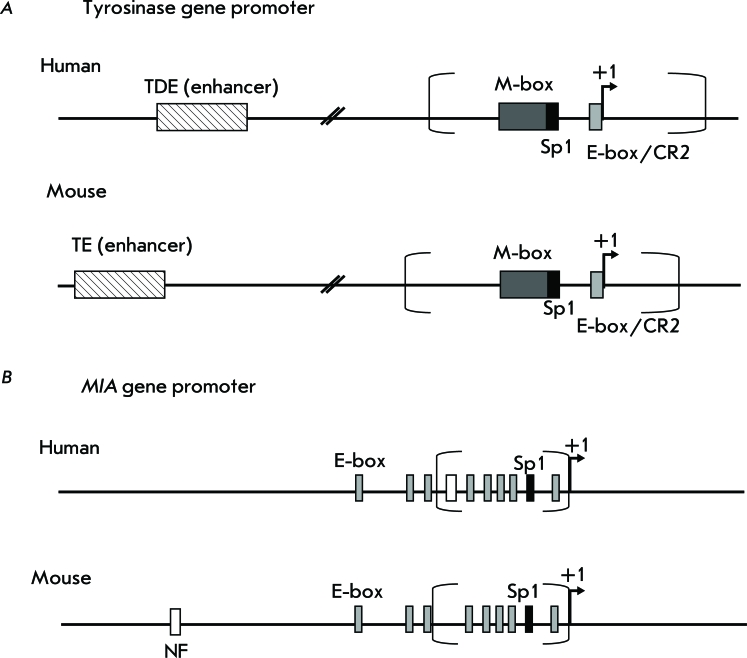
Schematic presentation of the *tyrosinase* and melanoma inhibitory activity ( *MIA* ) promoters. A – scheme of *tyrosinase * promoter, B – scheme of *MIA * promoter. The rectangle with diagonal hatching indicates an enhancer, open rectangle indicates the binding site of the transcription factor NF-κB, dark-gray rectangle – the M-box, light-gray rectangle – the E-box, black rectangle – the binding site of the Sp1 factor, a loci corresponding to the minimal promoter is given in parentheses. The arrow shows the transcription start site.

The Tyrex2 promoter that contains the human *TYR* core promoter and the tandem that consists of two mouse tyrosinase gene enhancers may serve as another example of using heterologous regulatory elements in order to enhance the *TYR* promoter [56]. When constructing the adenoviral vector, this promoter was used to control the activity of the *PNP* suicide gene, which can convert prodrug 6-methylpurine deoxyribonucleoside into a highly toxic purine-base 6-methylpurine [57]. After treating melanoma cells with adenovirus Tyrex2-PNP and introducing the prodrug, approximately 90% of the cells died. In nonmelanoma cell lines, there was a cytotoxic effect only when the nonspecific constitutive CMV promoter was used [56].

The reporter gene of chloramphenicol acetyltransferase (CAT) and therapeutic genes *HSVtk* and *DT-A * (diphtheria toxin A-chain) were used to compare the activity of the mouse *Tyr* gene promoters and human *MIA* gene promoters and their combinations with one or several enhancers of the mouse tyrosinase gene [22]. Promoters of genes encoding *Tyr* , *MIA* , and various combinations of them with one or several enhancers of the mouse tyrosinase gene provided a specific expression of both the *CAT* reporter gene and therapeutic genes *HSVtk* and *DT-A * in melanoma cell lines. It was demonstrated that the binding of several enhancers of the tyrosinase gene to the *MIA* promoter considerably enhances the specific activity of the *MIA* promoter in melanoma cell lines; the effect of mouse enhancers is stronger by an order of magnitude in comparison with that from human *TYR* enhancers, similar to the case of using the tyrosinase gene promoter. The strongest effect in both cases was observed when using constructs containing three or four mouse enhancers simultaneously [22].

The recombinant adeno-associated vector, in which the suicide gene is placed under the *MIA* promoter linked to the tandem consisting of four enhancer elements of the mouse tyrosinase gene, was described in [58]. The constructs with a full-size *MIA* promoter (1386 bp) and the minimal *MIA* promoter (493 bp) that is sufficient for maintaining the specific transcription in melanoma cells have been studied ( *Fig. B* ). It was shown that the constructs containing only *MIA* promoters have a low transcriptional activity only in melanoma cell lines [58]. The transcriptional potential of a construct with tyrosinase gene enhancers and the minimal *MIA* promoter was somewhat lower than that of the construction with the full-size promoter. The inhibition effect of melanoma cell growth with the use of *MIA* promoters with four tyrosinase gene enhancers is only slightly lower to the effect of the construct with the CMV-promoter; however, the latter does not provide the selectivity of transgene expression [58].

The given results demonstrate that the use of various combinations of melanoma-specific promoters and enhancers provides a high level of transgene expression in melanoma cells and can resolve the specificity problem for the gene therapy of the specified disease [50, 52, 56]. The *MIA* gene promoter is of special interest, since this gene, as opposed to the *TYR* gene, is expressed only in malignant melanoma cells but not in other cells of the melanocyte lineage. Thus, the regulatory elements of this gene may combine both tissue-specific and tumor-specific properties. However, the MIA gene promoter has still not been adequately studied as a candidate for the gene therapy of melanoma; almost nothing is known about the potential of the *MC1R * gene promoter.

## PROMOTERS SPECIFICALLY ACTIVE IN CELLS OF MELANOMA AND OTHER TUMORS 

Another approach that makes it possible to specifically control transgene expression in melanoma cells is the use of promoters that are specific not only in melanoma, but in other tumor cells as well. The examples of such tumor-specific promoters (TSP) include promoters of the genes *TERT* , *Cox-2, CXCR4* , and  *BIRC5* , for which overexpression of the genes controlled by them in numerous tumor types and the absence or a minimal expression in the normal tissues is typical.

The *TERT * gene encodes the catalytic subunit of human telomerase. This gene is active during the embryonic development and in tumor cells (in approximately 85% of all cases), whereas the expression of *TERT * is suppressed in the overwhelming majority of normal cells in the organism [59]. The level of *TERT * expression is increased for superficial spreading melanoma [60], which is believed to prevent the cells from entering apoptosis.

Cyclooxygenase 2 (Cox-2) is the inducible isoform of Cox-1, which cannot be detected in most normal tissues, as opposed to Cox-1 [61]. Expression of the *Cox-2 * gene is closely associated with cancerogenesis and the progression of certain types of intestinal neoplasias and tumors of epithelial origin [62, 63]. The *Cox-2 * gene is expressed at a high level in melanoma cells and is not expressed in nevus and in the normal epithelium of the gene [64].

The expression of the α-chemokine CDF-1receptor – *CXCR4 * gene is typical for breast cancer cells and virtually cannot be detected in the normal breast epithelium [65]. Overexpression of the CXCR3 and CXCR4 receptors was demonstrated in melanoma cells as well. It is assumed that receptors play a significant role upon melanoma invasion by modulating cell mobility, proliferation, and survival [66].

Survivin encoded by the *BIRC5 * gene belongs to the group of apoptosis-inhibitory proteins; it plays an important role in the growth and progression of tumors of various types [67]. *BIRC5 * is expressed in embryonic and fetal tissues [68], many types of neoplasias, including melanoma [69–71], and cannot be detected in differentiated adult tissues [72].

Tumor-specific promoters can be used within the conditionally replicating adenoviruses (CRAds) that were described above to achieve the oncolytic effect. Thus, the *TERT * promoter was used instead of the *E1A* gene promoter for transcriptional control of adenovirus replication. Moreover, this construct contained the apoptin gene under the strong constitutive CMV promoter [73]. Apoptin is a viral protein which specifically induces the apoptosis of cancer cells [74]. Thus, a system is constructed possessing “double” tumor-specificity, which is determined by the *TERT* promoter (activated in tumor cells) and apoptin (which has a selective effect on tumor cells). When cells were infected with Ad-TERT-Apoptin viruses, the growth of melanoma cells (line A375 and B16) was suppressed, resulting in apoptosis, whereas the normal epidermal melanocytes were protected from this effect [73]. Moreover, the reduction of lung metastases upon intratumoral and systemic administration of the Ad-TERT-Apoptin construct was demonstrated on a model of mouse metastatic melanoma. When using this system, a higher mouse survival rate was also observed [73].

The potential benefits of using other tumor-specific promoters in melanoma therapy were assessed by determining the activity of the luciferase reporter gene, when it is transcriptionally controlled by promoters of the *Cox-2, CXCR4* , and *BIRC5 * genes. Recombinant adenoviruses containing one of the TSP promoters and the luciferase gene controlled by them, instead of the Е1region, were used as a vector [75]. The luciferase activity was measured in four melanoma cell lines (Mel-624, A375M, SK-MEL-28 и MeWo) and in normal epithelial melanocytes (HEM) [75]. The *CXCR4 * gene promoter had no required specificity; its activity in the normal melanocytes was even higher than in melanoma cells [75]. Earlier, the transcriptional activity of the *Cox-2 * promoter that was inactive in primary melanocytes had been revealed in melanoma cell lines [76]. However, the activity of the *Cox-2 * gene promoter varies considerably depending on the cell line type [75]. The highest specific activity was demonstrated by the survivin gene promoter. Moreover, its activity in the normal melanocytes was considerably lower than it was in melanoma cells [75]. It has been recently shown that upon using the survivin gene promoter to control the expression of the iodide simporter ( *NIS* ) gene, the cells of the melanoma line A375 acquire the ability to uptake radioactive iodine-131, which has a negative effect on their survival [77]. Meanwhile, normal fibroblasts of human tooth pulp transfected by the same construct neither absorb iodine nor die. Thus, the survivin promoter was the optimal one for melanoma therapy among the tumor-specific promoters under comparison.

The activity of most tumor-specific promoters is lower than that of constitutive strong promoters, such as the SV40 and CMV promoters [75, 78, 79]. The activity of even relatively strong tumor-specific promoters varies considerably depending on the type of cancer cells. In different tumor cell lines, the activity of the survivin gene promoter varies from 0.3 to 16% of the activity of the CMV promoter [80–82], while the efficacy of the performance of the *TERT * promoter may differ by up to 20 fold [83].

The use of promoters that have a certain tissue-specific activity allows one to solve a number of problems associated with the nonspecific toxicity of the delivery vector. Thus, adenoviral vectors have considerable restrictions caused by the low efficacy of the transduction of melanoma cells, due to the low concentration or absence of the coxsackievirus and adenovirus receptors (CAR) that mediate cell transduction on melanoma cells [84]. The introduction of high doses of adenoviruses had a negative effect on the organism in general. Constructs of AdRGD adenoviruses that possess tropism towards α _V_ -integrins and transduce melanoma cells more efficiently as compared with the standard adenoviruses have been created [85]. Nevertheless, the systemic introduction of such adenoviral constructs resulted in nonspecific transduction and death of normal cells. The problem was solved by using specific promoters. AdRGD adenoviruses containing the *HSVtk * suicide gene under the transcriptional control of the tumor-specific promoter *TERT * or melanoma-specific promoter Tyrex2, instead of the standard nonspecific CMV promoter, turned out to be promising for melanoma therapy [86]. A decrease in the size of mice tumors was observed upon intratumoral introduction of AdRGD-TERT-HSVtk or AdRGD-Tyrex2-HSVtk, after subsequent administration of ganciclovir . The same effect is achieved by the introduction of low doses of nonspecific AdRGD-СМV-HSVtk; however, in this case increasing the dose of the vector resulted in weight loss and hepatotoxicity in the mice [86]. On the other hand, even an intravenous introduction of high doses of the AdRGD-TERT-HSVtk or AdRGD-Tyrex2-HSVtk vector does not cause toxic damage to the liver. Thus, the suppression of the nonspecific cytotoxicity of adenoviruses in normal nontumor cells is achieved via the use of specific promoters [86].

The size of the promoter plays a significant role in the design of efficient gene therapy agents, since many vectors are characterized by their limited capacity. Thus, it has been demonstrated that in retroviral vectors containing long promoter modules, the viral titer typically decreases when the size of the promoter introduced is increased [87]. However, a large number of short promoters are either very weak or lose their tissue specificity; therefore, the possibility of constructing short and specific promoters that would possess sufficient transcriptional potential is a considerable task. Construction of synthetic and/or double (chimeric) promoters may become a method that could allow to overcome these limitations.

## CONSTRUCTION AND OPTIMIZATION OF SYNTHETIC and DOUBLE PROMOTERS POSSESSING TISSUE-SPECIFIC ACTIVITY 

The controlling elements of the known promoters that have been characterized are used to construct *de novo* specific promoter modules. For instance, artificial promoters based on the elements of the promoters of the human tyrosinase and α-fetoprotein (AFP1) genes possessing strong and specific expression in melanoma cell lines were constructed [88]. As previously mentioned, the tyrosinase promoter contains the M-box, the conservative element that is common to melanocyte-specific promoters [89]. This element was used in combination with the elements from the 5’-region of the *AFP1 * promoter – the GRE element, which is specific for the cell cycle and the AP1-binding element. Several efficient melanocyte-specific promoters were obtained upon different combinations of one or several copies of fragments of the tyrosinase and α-fetoprotein promoters – the M-box, AP1 and GRE elements. These promoters were selectively active in the B16 melanoma line, but not in the HeLa cell line [88]. The length of the artificial constructs was not more than 300 bp; the promoter consisting of three GRE, three AP1 elements, and two M-boxes was the most efficient one. It was ascertained that if the number of regulatory elements of the promoter in the chimeric construct is higher than eight units, a loss in promoter specificity is observed [88]. It seems that the activity of synthetic promoters is dependent both upon the number of regulatory elements and upon the vector. The optimal number of regulatory elements has to be selected in each case. For example, it was shown in the above-mentioned study by Rothfels *et al* . [22] that it is sufficient to bind four copies of the enhancers of the mouse tyrosinase gene in order to increase the activity of both the *MIA* and *TYR* promoters.

Another approach to constructing specific promoters consists in the construction of chimeric or double promoters. 

As previously mentioned, most tumor-specific promoters show lower activity in comparison with constitutive strong promoters, such as the promoters of the SV40 and CMV viruses. One of the approaches that help to resolve the problems related to the efficacy of tumor-specific promoters is the use of hybrid double promoters: (i) one of them being tumor-specific, while the second is a strong nonspecific promoter; (ii) each promoter being tumor-specific. The double promoters described exhibit a higher activity in tumors of a certain type in comparison with natural promoters. 

The chimeric promoter CMV-hTERT is an example of the first construct [90]. The chimeric construct was obtained on the basis of the promoter of the human telomerase reverse transcriptase (hTERT) gene and the minimal CMV promoter, which is characterized by a higher activity in comparison with the nonmodified * hTERT* promoter, while it retains tumor specificity.

Double tumor-specific promoters have also been described [91, 92]. In order to increase the efficacy of expression of therapeutic genes in small lung cancer cell (SCLC), a chimeric double promoter based on promoters of the *hASH1* and *EZH2 * genes with a high level of expression in SCLC cells was obtained. The activity of the double chimeric promoter was higher than the activities of the corresponding single promoters by up to 1–8 fold, depending on the cell line of SCLC [92].

In another study, a high level of expression of apoptosis activator *tBid * in breast cancer cells was achieved via the use of a hybrid promoter consisting of promoters of the human survinin gene and the gene encoding glycoprotein mucin; its expression increased in breast cancer cells [91]. Thus, the use of double promoters permits one to provide a high level of expression of the therapeutic gene in tumor cells, retaining specificity towards cancer. The use of double tumor-specific promoters facilitates the construction of more universal gene therapeutic constructs: i.e., constructs that provide the expression of the therapeutic gene in many types of cancer cells. For instance, a vector bearing two *DT-A* gene fragments controlled by the promoters of the *IGF2-P4* and  *H19 * genes was constructed [93]. The introduction of this vector to cells of several lines of urinary bladder cancer ensured gene expression in all lines, whereas the *DT-A * gene controlled by one of the promoters, *IGF2-P4* or *H19* , exhibited activity not in all the lines of tumor cells that were used.

No systems of double promoters containing melanoma-specific promoters have been described yet. However, the construction of systems based on melanoma-specific promoters, such as promoters of the *MIA* and *TYR * genes, could provide a universal, highly efficient, and specific expression of the therapeutic gene in melanoma cells.

## CONCLUSION 

Melanoma treatment is associated with a number of difficulties, including the high resistance of melanoma cells and early metastazing, which determine the unfavourable prognosis. The necessity to affect the metastatic loci disseminated over the entire organism requires the systemic administration of antimelanoma agents, which involves a certain risk that other cells of the organism can be affected. Gene therapy, which has known rapid development, offers new methods that allow to increase the specificity of the effect on melanoma cells whilst simultaneously decreasing the probability of damaging healthy cells. The use of melanoma-specific promoters makes it possible to specifically affect melanoma cells. These methods have recently gone into the stage of development and are permanently being improved to find the most efficient solutions, starting with the selection of optimal regulatory elements, the designing of constructs based on these elements, and ending with the search for new vectors, with both natural viruses or artificially constructed systems for packing the genetic material being used as such vectors [94, 95]. One can hope that a simple and efficient system for the elimination of melanomas and its metastases will be designed.
